# Connective Tissue Degeneration: Mechanisms of Palmar Fascia Degeneration (Dupuytren’s Disease)

**DOI:** 10.1007/s40610-016-0045-3

**Published:** 2016-07-14

**Authors:** S. Karkampouna, M. Kreulen, M. C. Obdeijn, P. Kloen, A. L. Dorjée, F. Rivellese, A. Chojnowski, I. Clark, Marianna Kruithof-de Julio

**Affiliations:** 1 Department of Urology, Leiden University Medical Center, Albinusdreef 2, Leiden, ZA 2333 The Netherlands; 2 Department of Plastic Surgery, Rode Kruis Ziekenhuis, Vondellaan 13, Beverwijk, 1942 LE The Netherlands; 3 Department of Plastic Reconstructive and Hand Surgery, Academic Medical Center, Meibergdreef 9, Amsterdam, 1100 DD The Netherlands; 4 Department of Orthopedic Surgery, Academic Medical Center, Meibergdreef 9, Amsterdam, 1100 DD The Netherlands; 5 Department of Rheumatology, Leiden University Medical Center, Albinusdreef 2, Leiden, 2333 ZA The Netherlands; 6 Centre for Experimental Medicine and Rheumatology, William Harvey Research Institute, Barts and The London School of Medicine and Dentistry, Queen Mary University of London, London, UK; 7 Institute of Orthopaedics, Norfolk and Norwich University Hospital, Norwich, UK; 8 Biomedical Research Centre, School of Biological Sciences, University of East Anglia, Norwich, UK; 9 Urology Research Laboratory, Department of Urology and Department of Clinical Research, University of Bern, Murtenstrasse 35, Bern, 3008 Switzerland

**Keywords:** Inflammation, Dupuytren’s, Fibrosis, Regeneration, Contracture

## Abstract

Dupuytren’s disease is a connective tissue disorder of the hand causing excessive palmar fascial fibrosis with associated finger contracture and disability. The aetiology of the disease is heterogeneous, with both genetic and environmental components. The connective tissue is abnormally infiltrated by myofibroblasts that deposit collagen and other extracellular matrix proteins. We describe the clinical profile of Dupuytren’s disease along with current therapeutic schemes. Recent findings on molecular and cellular parameters that are dysregulated in Dupuytren’s disease, which may contribute to the onset of the disease, and the role of resident inflammation promoting fibrosis, are highlighted. We review recent literature focusing on non-myofibroblast cell types (stem cell-like cells), their pro-inflammatory and pro-fibrotic role that may account for abnormal wound healing response.

## Introduction

Wound repair and tissue regeneration after injury is widely accepted to occur during the adult life of large mammals. In humans, liver regeneration after partial hepatectomy [1] or gum regeneration [2] is a complete and restorative process. However, skin wounds, incisions or excisions lead to formation of scar tissue, which does not resolve for a long term, thus, the cell-mediated tissue regeneration is incomplete while scar tissue persists. Embryonic skin wounds lead to scar-free, completely regenerated tissues, while the majority of mechanical injuries during adult life lead to scar tissue formation [3]. Thus, there might exist a “co-dependent” link between tissue regeneration, cell replenishment and scarring. The early phases of the wound healing response are dependent on inflammation and fibrogenesis, recruitment of platelets, immune cell and fibroblast invasion, pro-inflammatory cytokine secretion, differentiation of fibroblasts to myofibroblasts and fibrin clot formation. If the damaging stimuli are repetitive, this will lead to persistent inflammation; higher levels of interleukins, tumor necrosis factor alpha (TNFα) and pro-fibrogenic transforming growth factor beta (TGFβ) and therefore scarring. It has been proposed that the same signals that regulate scar-free embryonic regeneration also regulate the adult wound healing response. These cellular processes might be controlled by the levels and/or localization of those same signals as well as of the (extra)cellular context, developmental stage, tissue specificity and repetitive versus acute injury.

In the case of Dupuytren’s disease (DD), although it is not clear whether its pathogenesis is of mechanical or biochemical nature, the net result is the same: excess production of matrix proteins and excessive accumulation of extracellular matrix (scarring) which changes tissue architecture and causes digital contraction. Perhaps we should re-evaluate DD not only as excess scarring but also as a condition of abnormal tissue regeneration.

In this review, we will discuss the recent research findings on DD focusing on the role of non-fibroblastic cell populations (immune cells, vascular cells or potential stem/progenitor cells).

## Clinical Problems of DD and Current Therapeutic Possibilities

**Fig. 1:**
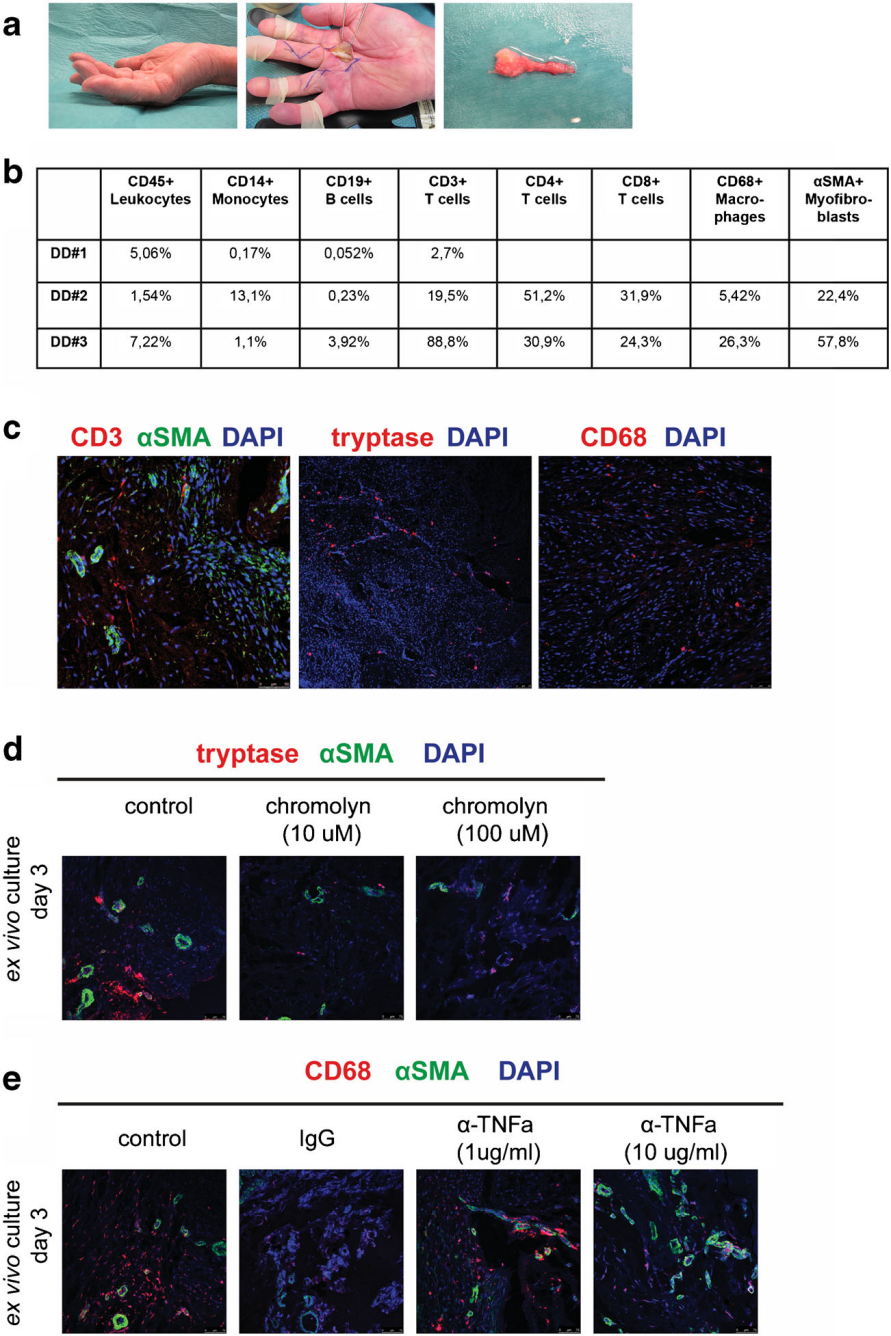
**a** Clinical presentation of Dupuytren’s disease; preoperative rigid contracture, surgical incision during palmar fasciectomy with prevalent collagen cord, resected nodule and cord specimen. **b** Immune cell types (leukocytes, monocytes, B and T cells) residing in nodules from DD patient material (FACS analysis, *N* = 3). **c** Immunofluorescence of CD3, alpha smooth muscle actin (αSMA), tryptase and CD68 expression in Dupuytren’s nodules. DAPI (nuclei). **d** *Ex vivo* culture of Dupuytren’s nodules and treatments with mast cell stabilizer chromolyn. Immunofluorescence for αSMA (myofibroblasts) and tryptase expression (mast cells). **e** *Ex vivo* culture of Dupuytren’s nodules and treatment with anti-TNFa antibody (golimumab) and control IgG. Immunofluorescence for αSMA (myofibroblasts) and CD68 expression (macrophages)

Dupuytren’s disease typically presents in the fourth and fifth decade of life with thickening and nodule formation of the affected fascial structures in the hand (Fig. 1a) [4].

Disease progression leads to longitudinally oriented cord-like structures that limit extension of the involved fingers and ultimately to metacarpophalangeal (MCP) and interphalangeal joint (PIP or DIP) contractures [5]. Younger patients often have a more aggressive disease progression. Most patients with a first presentation of DD do not have pain or functional disability and require no treatment. A small extension deficit of the MCP joints might lead to a positive “tabletop test” (the hand cannot lie flat on a tabletop), but few patients experience functional limitations. The indications for treatment are governed by functional loss and progression and may be subject to local healthcare system guidelines. An extension deficit greater than 30° may lead to contracture of the accessory collateral ligaments and the palmar plate of the PIP joint. PIP joint contractures are generally regarded as more difficult to treat than MCP contractures because of the secondary joint contracture and weakening of the extensor mechanism caused by palmar fibrosis.

A wide range of treatment options are available [6] generally involving mechanical release or excision of excessive fibrotic tissue. However, recurrence rates are high, ranging from 8 to 66 % (average 33 %) [7]. The surgeon and patient should be aware that there is no curative treatment for the disease.

## Non-operative Treatment

Non-operative treatments such as physiotherapy, splinting and local radiotherapy may affect disease progression, but long-term efficacy is unclear.

## Minimally Invasive (Percutaneous) Treatment

Minimally invasive (or percutaneous) techniques have become more popular as can be performed in the outpatient setting. Principally, they involve either collagenase injections or needle fasciotomy. The goal is to rupture palpable cords causing digital flexion contracture. Local triamcinolone acetonide injections do not release DD contractures.

Collagenase is an enzyme solution (derived from *Clostridium histolyticum*), which is injected directly into the DD cord. The cord will weaken due to enzymatic digestion and rupture when manipulated over the next few days. This technique (FDA-approved [8]) tends to be effective for MCP contractures, though with certain complications, and is considered a promising alternative for less severe contractures. It is shown to be of particular value in MCP contractures [9]. However, recurrences are reported and complications of oedema, tendon rupture, pain and lymphadenopathy are described [5].

Percutaneous needle fasciotomy has been popularized in recent decades. Cutting the cord with a needle is effective for solitary central cords in the palm of the hand to release MCP contractures. More distal, the effectiveness of the release decreases and the risk of iatrogenic neurovascular damage increases. Also, high recurrence rates up to 60 % within 3 years are reported [10]. These less invasive techniques may be a useful tool in the sicker patients with co-morbidities who cannot undergo surgery or in those who want immediate improvement.

## Surgical Techniques

Surgical excision of diseased tissue is called fasciectomy and can vary from limited to radical excision. For skin incisions, there are multiple options ranging from midline Y-V advancement flaps, Z-plasties and zigzag (Bruner-type) incisions [11]. Radical fasciectomy has high complication rate without a significantly decreased recurrence rate. Dermofasciectomy involves removal of the diseased fascia including the overlying skin. This technique, if used radically, may reduce long-term recurrence rates. Smaller or “firebreak” skin grafts probably do not improve recurrence rates over fasciectomy alone. Partial fasciectomy is considered the gold standard of surgical treatment for functionally disabling DD [11].

After removal of the diseased tissue (fasciectomy), additional procedures might be necessary to correct capsular joint contractures (PIP joint capsulolysis) or to reconstruct a skin defect (local skinflap or graft). Postoperative splinting of the hand may not improve medium-term outcome [5]. Complications of open surgery include delayed wound healing, skin flap necrosis, digital nerve and vessel injury, joint stiffness, hematoma and pain issues [12]. Complex regional pain syndrome (CRPS) can be a devastating complication of surgery prolonging recovery and requiring long-term hand therapy support and chronic pain treatments [13].

## Molecular and Cellular Alterations in DD Degeneration

The main characteristic of DD is accumulation of extracellular matrix proteins, which form an abnormal connective tissue of the palmar fascia mainly containing collagen (mostly collagen type I and type III) and myofibroblasts [14]. Several studies have implicated the TGFβ and WNT pathways as drivers of fibrosis in DD (reviewed in [15–17]). Expression analyses in DD (by e.g. microarray or q-RT-PCR) [18–27] have shown that these key pathways are indeed modulated, but do not always yield the same results: either different set of hits or different mode of expression (downregulation versus upregulation). Considering the heterogeneity among individuals and additional factors including biopsy material or comparison with carpal tunnel-derived fascia palmaris or “normal” adjacent tissue from DD patients, as well as the derivation of tissue fibroblasts, there is high variability in the outcomes.

A recent genome-wide analysis study using exon arrays indicated a variety of genes that are differentially expressed in DD patient fibroblasts compared with control (thigh skin punch form unaffected individuals) fibroblasts [28]. Among the list of genes are ECM and tissue re-modelling genes suggesting aberrant matrix synthesis and turnover. Top hits are the matrix metalloproteinase-1 (MMP-1), MMP-3 and MMP-16, which have decreased expression in DD fibroblasts, though other studies have implicated MMP-2 and MMP-14 [29]. MMP-1, MMP-14 and to some extend MMP-2 are collagen-degrading enzymes, whilst MMP-3 and MMP-16 activate such enzymes. ADAM15, ADAMTS10, ADAMTS2 and ADAMTS3 showed increased expression in DD patient samples. ADAMTS2 and ADAMTS3 are procollagen propeptidases, involved in collagen biosynthesis. More than 20 collagen genes showed upregulated expression in DD especially those of the COL1, COL3, COL4 and COL5 cluster.

Regulation of cell surface proteins involved in interaction with ECM, such as the integrin family, is disrupted, showing either significantly higher (ITGA11) or lower expression (ITGA2, ITGA6 and ITGA4). Members of the TGFβ and WNT pathway are also dysregulated e.g. follistatin, BMP4, inhibin subunit INHBA, WNT2, frizzled 4, and RSPO3. A previous genome-wide association study has shown several genes of the WNT signalling pathway to be dysregulated (WNT2, WNT4, WNT7B, RSPO2, SFRP4, SULF1) [27]. Further characterization has indicated decreased WNT2 and increased b-catenin and WNT7B in the DD nodules along with increased ACTA2 (α-smooth muscle actin, a myofibroblast marker) and COL1A1 and COL3A1 expression [30••].

Novel players have been implicated in the pathogenesis of DD such as Wilm’s tumour protein-1 (WT1) [31•] and YAP [32•]. YAP1, a member of the Hippo pathway, which has recently been shown to promote differentiation of fibroblasts into myofibroblasts, potentially acts downstream of TGFβ [32•].

microRNAs (miRNAs) are implicated in many biological processes and have been associated with several diseases; however, few studies have investigated the potential role of miRNAs in DD fibrosis. The first microarray studies identified unique profiles of miRNAs in control versus DD fascia palmaris [33, 34]. Mosakhani et al. reported that some miRNAs (e.g. miR29-C, miR29-130b, miR29-101) are predicted to regulate both the WNT and TGFβ pathways [34]. RNA sequencing analysis of DD and the control fascia material shows a unique enrichment of over 70 miRNAs in the DD and a distinct, smaller subset enriched in control fascia [35]. Target prediction analysis indicated that anti-fibrotic miRNAs targeting collagen mRNAs, which are present in the normal fascia, are in fact depleted in DD patient material [35]. Thus, these studies add to our understanding of DD pathogenesis.

## Inflammation and Fibrosis

The majority of knowledge on aspects of the wound healing response has derived by studies of acute skin injuries. Resident and inflammatory cells (e.g. mast cells, leukocytes) release growth factors, proteases and prostaglandins that are essential for removal of damaged epithelial cells, protection from infectious factors and activation of fibroblasts into myofibroblasts that form the fibrous scar tissue and have mechanical properties to mediate wound closure. Although acute tissue injury is resolved completely, repetitive chronic injury, the addition of other factors such as age or diabetes or chronic inflammation have the potential to interfere with the correct remodelling of tissue and are contributing factors to persistent scarring (e.g. hypertrophic scars) [36, 37]. For instance, macrophage and leukocyte-depleted transgenic mice (PU.1 null) have rapid skin wound repair with reduced fibrosis [38]. The mechanism by which inflammation influences fibrosis remains elusive. Similarly to skin wound repair, connective tissue diseases such as DD or Peyronie’s disease are likely to be affected by inflammation. Despite the first study that reported macrophages and leukocytes around the DD nodules [39], the role of immune cells in DD fibrosis had not been characterized until recently. A limitation that accounts for this was the overlapping recognition of fibroblasts by anti-macrophage antibodies (Mac-3, CD68, MHC class II, CD45), which may have led to misidentification of pure fibroblast and macrophage cell populations [40].

High levels of inflammatory cytokines were detected in tissue from DD patients along with CD68+ monocytes and classically activated M1 (pro-inflammatory) and alternatively activated M2 (regenerative) macrophages [41]. The same study showed that pro-inflammatory factor TNFα promotes DD fibrosis, although IL-6 and IL-1b did not have the same effect, via activation of WNT signalling [41]. Bianchi et al. have also reported increased expression of IL-6 and IL-1b cytokines in DD as well as presence of CD68-positive cells [42]. The pro-fibrogenic factor TGFβ, released by platelets, fibroblasts and macrophages, also mediates inflammation-related signalling (such as p38 activation) also in DD fibroblasts [45, 44].

Our unpublished observations demonstrate the presence of a small number of immune cell types (leukocytes, monocytes, B and T cells) residing in nodules from DD patient material (Fig. 1b). Immunofluorescence of fixed material immediately after surgical removal of nodules confirmed the presence of CD3-positive T cells along with CD68-positive macrophages (Fig. 1c) similar to the study of Verjee et al. In addition, we detected tryptase, a well-known specific alpha marker of mast cells (Fig. 1c). Mast cells are granulated tissue-resident cells of the innate immunity, best known for their involvement in allergic disorders, but also playing a role in several autoimmune conditions [45], where they can have both pro- and anti-inflammatory/immunomodulatory effects [46, 47]. In the context of Dupuytren’s inflammation, mast cell activation could contribute to the modulation of the inflammatory response leading to fibrosis. For example, tryptase-initiated signalling influences neutrophil and monocyte recruitment, muscle tissue regeneration [48], fibroblast proliferation [49, 50] and activation of latent TGFβ [51]. Interestingly, the number of mast cells has been shown to be increased in Dupuytren’s contracture in comparison with normal fascia tissue [52]. Inhibition of mediator release by the mast cell stabilizer compound chromolyn (chromoglicic acid) is used as anti-allergy treatment for asthma, conjunctivitis and food allergies [53]. As a proof of principle, we tested the effect of inhibition of mast cells *ex vivo* and their potential influence upon fibroblasts on our *ex vivo* human tissue culture method [54••, 55]. We have exposed DD nodule-derived slices to chromolyn for 72 h; our preliminary data suggest a decrease in tryptase and alpha smooth muscle actin (αSMA) expression (Fig. 1d). Co-labelling of CD68 and αSMA in DD specimens indicates the presence of macrophages around the microvessel clusters (Fig. 1e, left panel), while *ex vivo* treatment of tissue with neutralizing TNFα antibody (golimumab) has little effect at the lowest concentration (1 μg/ml) but leads to reduced CD68 expression when used at a higher concentration (10 μg/ml).

A recent study has characterized the immune response in a large subset of DD specimens and, similar to our unpublished observations, has detected the infiltration of DD tissue by several immune cells. After extensive characterization of T cells and cytokine profiling, the authors suggest that T cells may contribute to the development of DD possibly via (auto)antigen-driven processes possibly due to microvascular damage [56••].

Overall, the fibrotic response in DD is being recognized as an immune-mediated response, with an important involvement of different immune cells. Our preliminary observations suggest that mast cells could also be involved in the inflammatory response leading to the development of fibrosis, an intriguing observation warranting additional investigations on the role of mast cells and other immune cells in DD.

## Contribution of Stem/Progenitor Cells in DD

The field of DD research is mainly focused on the mechanisms of deregulated proliferation of myofibroblasts and their matrix-producing properties. However, this may be at the end stage of the disease and not necessarily during its onset. The switch to excessive fibrosis may be indeed controlled by other cell types such as the infiltrating immune cells, vascular smooth cells, endothelial cells, pericytes [57], fibrocytes originating from the bone marrow or possibly multilineage progenitors that give rise to myofibroblasts [58] (mesenchymal stem/stromal cells). Pericytes, the endothelium-covering cells, have been attributed with stem cell properties in several organs [59], which is likely to be the case also in DD fibrosis. Vessel structures that contain high levels of laminins, a key basal membrane component of the connective tissue, facilitate proliferation of myofibroblasts evident by proliferating centres in the vicinity of these vessels [60••]. Endothelial cell and mesenchymal stem cell (MSC)-enriched protein CD105 (type III receptor, endoglin) has been found to be expressed near these proliferation centres. Vessel structures in DD specimens appear abnormally large or with fused vessels and are located distinctly from the myofibroblast-enriched area. It is highly likely that these structures are sweat glands from subcutaneous dermis that seem to be encapsulated within the fibrotic nodules as also suggested by Viil et al. The presence of stem cells has been reported in the vicinity of cutaneous sweat glands [61]; thus, we hypothesize that similar stem/progenitor cell populations may exist in DD. Given the high proliferative properties of these myofibroblasts, such an assumption seems probable; however, detailed investigations are needed.

Cell replacement treatments using MSCs for organ fibrosis, such as liver [62], lung [63] or heart [64], have been attempted in many studies in order to improve cell replenishment and tissue regeneration. MSCs are multipotent stromal cells which differentiate into distinct cell lineages: osteoblasts, chondrocytes, adipose cells, muscle cells as well as tenocytes, skin cells and differentiated stromal cells of connective tissue (fibroblast phenotype) [65]. However, the ability of MSCs to give rise to fibroblasts is often overlooked, along with the subsequent effects on exacerbating instead of ameliorating fibrosis. MSCs may differentiate into highly specified fibroblastic populations such as inflammatory fibroblasts or myeloid fibroblasts. In DD, palmar fascia tissue recent studies have reported and characterized the presence of resident MSCs or adipose stem cells [66••, 67••], or a stem cell-like subpopulation of Thy1 (CD90)-positive cells has been identified [68••]. Cell-cell contact of DD myofibroblasts with adipose stem cells resulted in inhibition of contractility and smooth muscle actin expression of myofibroblasts [69••]. A recent study has identified an increased resident and circulating fibrocyte population in DD tissue compared to control tissue [70••]. Fibrocyte characterization showed that these cells are of the mononuclear cell lineages sharing properties with fibroblasts and mesenschymal stromal cells (CD45RO, 25F9 and MRP8/14). Treament of fibrocytes with serum amyloid P and FDA-approved Xiapex collagenase inhibited the expansion of fibrocytes in vitro, a promising finding regarding the extended use of Xiapex [70••].

## Conclusions

In this review, we have discussed the clinical problems associated with DD and the current therapeutic tools currently available. In addition, we have reviewed recent publications on studies regarding novel regulator genes or cellular processes involved in DD pathogenesis. We have discussed how fibrosis and tissue regeneration are inter-dependent processes, for instance deregulated tissue regeneration or “re-cellularization” leads to overstimulation of fibrosis. One of the underlying factors regulating fibrosis and regeneration is inflammation, while the role of inflammation in DD is only beginning to be unravelled. The latest research studies introduce the possibility that DD may be an autoimmune disease, which however requires more investigation. Given the high demand for safe and effective anti-fibrotic drugs for DD, we propose that novel compounds that inhibit both myofibroblasts and immune cells (mast, B and T cells) or a combination of anti-fibrotic and anti-inflammatory compounds are promising candidates. From a different point of view, we discuss the possibility of resident stem/progenitor cells to be a pool for myofibroblasts or inhibit their fibrogenic activity based on recent publications. The potential of MSC differentiation to the fibroblast lineage or stromal precursor cells has not yet been characterized in DD.

## References

[1] Michalopoulos GK (2007). Liver regeneration. J Cell Physiol.

[2] Park YJ, Cha S, Park YS (2016). Regenerative applications using tooth derived stem cells in other than tooth regeneration: a literature review. Stem Cells Int.

[3] Ferguson MW, O’Kane S (2004). Scar-free healing: from embryonic mechanisms to adult therapeutic intervention. Philos Trans R Soc Lond B Biol Sci.

[4] Rayan GM (2007). Dupuytren disease: anatomy, pathology, presentation, and treatment. J Bone Joint Surg Am.

[5] Eaton C (2014). Evidence-based medicine: Dupuytren contracture. Plast Reconstr Surg.

[6] Desai SS, Hentz VR (2011). The treatment of Dupuytren disease. J Hand Surg Am.

[7] Van Giffen N, Degreef I, De Smet L (2006). Dupuytren’s disease: outcome of the proximal interphalangeal joint in isolated fifth ray involvement. Acta Orthop Belg.

[8] Gilpin D, Coleman S, Hall S, Houston A, Karrasch J, Jones N (2010). Injectable collagenase Clostridium histolyticum: a new nonsurgical treatment for Dupuytren’s disease. J Hand Surg Am.

[9] Hurst LC, Badalamente MA, Hentz VR, Hotchkiss RN, Kaplan FTD, Meals RA (2009). Injectable collagenase Clostridium histolyticum for Dupuytren’s contracture. N Engl J Med.

[10] Watt AJ, Curtin CM, Hentz VR (2010). Collagenase injection as nonsurgical treatment of Dupuytren’s disease: 8-year follow-up. J Hand Surg Am.

[11] Rodrigues JN, Becker GW, Ball C, Zhang W, Giele H, Hobby J (2015). Surgery for Dupuytren’s contracture of the fingers. Cochrane Database Syst Rev.

[12] Mavrogenis AF, Spyridonos SG, Ignatiadis IA, Antonopoulos D, Papagelopoulos PJ (2009). Partial fasciectomy for Dupuytren’s contractures. J Surg Orthop Adv.

[13] Bulstrode NW, Jemec B, Smith PJ (2005). The complications of Dupuytren’s contracture surgery. J Hand Surg Am.

[14] Bazin S, Le Lous M, Duance VC, Sims TJ, Bailey AJ, Gabbiani G (1980). Biochemistry and histology of the connective tissue of Dupuytren’s disease lesions. Eur J Clin Invest.

[15] Tomasek JJ, Gabbiani G, Hinz B, Chaponnier C, Brown RA (2002). Myofibroblasts and mechano-regulation of connective tissue remodelling. Nat Rev Mol Cell Biol.

[16] Bowley E, O’Gorman DB, Gan BS (2007). β-catenin signaling in fibroproliferative disease. J Surg Res.

[17] Nunn AC, Schreuder FB (2014). Dupuytren’s contracture: emerging insight into a Viking disease. Hand Surg.

[18] Shih B, Watson S, Bayat A (2012). Whole genome and global expression profiling of Dupuytren’s disease: systematic review of current findings and future perspectives. Ann Rheum Dis.

[19] Qian A, Meals RA, Rajfer J, Gonzalez-Cadavid NF (2004). Comparison of gene expression profiles between Peyronie’s disease and Dupuytren’s contracture. Urology.

[20] Rehman S, Salway F, Stanley JK, Ollier WER, Day P, Bayat A (2008). Molecular phenotypic descriptors of Dupuytren’s disease defined using Informatics analysis of the transcriptome. J Hand Surg Am.

[21] Zhang AY, Fong KD, Pham H, Nacamuli RP, Longaker MT, Chang J (2008). Gene expression analysis of Dupuytren’s disease: the role of TGF- β2. J Hand Surg Eur Vol.

[22] Johnston P, Chojnowski AJ, Davidson RK, Riley GP, Donell ST, Clark IM (2007). A complete expression profile of matrix-degrading metalloproteinases in Dupuytren’s disease. J Hand Surg Am.

[23] Lee LC, Zhang AY, Chong AK, Pham H, Longaker MT, Chang J (2006). Expression of a novel gene, MafB, in Dupuytren’s disease. J Hand Surg Am.

[24] Shih B, Wijeratne D, Armstrong DJ, Lindau T, Day P, Bayat A (2009). Identification of biomarkers in Dupuytren’s disease by comparative analysis of fibroblasts versus tissue biopsies in disease-specific phenotypes. J Hand Surg Am.

[25] Pan D, Watson HK, Swigart C, Thomson JG, Honig SC, Narayan D (2003). Microarray gene analysis and expression profiles of Dupuytren’s contracture. Ann Plast Surg.

[26] Furniss D, Dolmans GH, Hennies HC (2011). Genome-wide association scan of Dupuytren’s disease. J Hand Surg Am.

[27] Dolmans GH, Werker PM, Hennies HC, Furniss D, Festen EA, Franke L (2011). Wnt signaling and Dupuytren’s disease. N Engl J Med.

[28] Forrester HB, Temple-Smith P, Ham S, de Kretser D, Southwick G, Sprung CN (2013). Genome-wide analysis using exon arrays demonstrates an important role for expression of extracellular matrix, fibrotic control and tissue remodelling genes in Dupuytren’s disease. PLoS One.

[29] Wilkinson JM, Davidson RK, Swingler TE, Jones ER, Corps AN, Johnston P (2012). MMP-14 and MMP-2 are key metalloproteases in Dupuytren’s disease fibroblast-mediated contraction. Biochim Biophys Acta.

[30.••] van Beuge MM, ten Dam E-JPM, Werker PMN, Bank RA (2015). Wnt pathway in Dupuytren disease: connecting profibrotic signals. Transl Res.

[31.•] Crawford J, Raykha C, Charles D, Gan BS, O’Gorman DB (2015). WT1 expression is increased in primary fibroblasts derived from Dupuytren’s disease tissues. J Cell Commun Signal.

[32.•] Piersma B, de Rond S, Werker PMN, Boo S, Hinz B, van Beuge MM (2015). YAP1 Is a driver of myofibroblast differentiation in normal and diseased fibroblasts. Am J Pathol.

[33] Satish L, LaFramboise WA, Johnson S, Vi L, Njarlangattil A, Raykha C (2012). Fibroblasts from phenotypically normal palmar fascia exhibit molecular profiles highly similar to fibroblasts from active disease in Dupuytren’s Contracture. BMC Med Genomics.

[34] Mosakhani N, Guled M, Lahti L, Borze I, Forsman M, Paakkonen V (2010). Unique microRNA profile in Dupuytren’s contracture supports deregulation of β-catenin pathway. Mod Pathol.

[35] Riester SM, Arsoy D, Camilleri ET, Dudakovic A, Paradise CR, Evans JM (2015). RNA sequencing reveals a depletion of collagen targeting microRNAs in Dupuytren’s disease. BMC Med Genomics.

[36] Wong VW, Paterno J, Sorkin M, Glotzbach JP, Levi K, Januszyk M (2011). Mechanical force prolongs acute inflammation via T-cell-dependent pathways during scar formation. FASEB J.

[37] Eming SA, Martin P, Tomic-Canic M (2014). Wound repair and regeneration: mechanisms, signaling, and translation. Sci Transl Med.

[38] Martin P, D’Souza D, Martin J, Grose R, Cooper L, Maki R (2003). Wound healing in the PU.1 null mouse—tissue repair is not dependent on inflammatory cells. Curr Biol.

[39] Andrew JG, Andrew SM, Ash A, Turner B (1991). An investigation into the role of inflammatory cells in Dupuytren’s disease. J Hand Surg Br.

[40] Inoue T, Plieth D, Venkov CD, Xu C, Neilson EG (2005). Antibodies against macrophages that overlap in specificity with fibroblasts. Kidney Int.

[41] Verjee LS, Verhoekx JSN, Chan JKK, Krausgruber T, Nicolaidou V, Izadi D (2013). Unraveling the signaling pathways promoting fibrosis in Dupuytren’s disease reveals TNF as a therapeutic target. Proc Natl Acad Sci U S A.

[42] Bianchi E, Taurone S, Bardella L, Signore A, Pompili E, Sessa V (2015). Involvement of pro-inflammatory cytokines and growth factors in the pathogenesis of Dupuytren’s contracture: a novel target for a possible future therapeutic strategy?. Clin Sci.

[43] Bujak M, Ratkaj I, Markova-Car E, Jurišić D, Horvatić A, Vučinić S (2015). Inflammatory gene expression upon TGF-β1-induced p38 activation in primary Dupuytren’s disease fibroblasts. Front Mol Biosci.

[44] Krause C, Kloen P, ten Dijke P (2011). Elevated transforming growth factor β and mitogen-activated protein kinase pathways mediate fibrotic traits of Dupuytren’s disease fibroblasts. Fibrogenesis Tissue Repair.

[45] Suurmond J, van der Velden D, Kuiper J, Bot I, Toes RE (2015). Mast cells in rheumatic disease. Eur J Pharmacol.

[46] Suurmond J, Rivellese F, Dorjee AL, Bakker AM, Rombouts YJ, Rispens T (2015). Toll-like receptor triggering augments activation of human mast cells by anti-citrullinated protein antibodies. Ann Rheum Dis.

[47] Rivellese F, Suurmond J, Habets K, Dorjee AL, Ramamoorthi N, Townsend MJ (2015). Ability of Interleukin-33- and immune complex-triggered activation of human mast cells to down-regulate monocyte-mediated immune responses. Arthritis Rheum.

[48] Duchesne E, Bouchard P, Roussel MP, Cote CH (2013). Mast cells can regulate skeletal muscle cell proliferation by multiple mechanisms. Muscle Nerve.

[49] Frungieri MB, Albrecht M, Raemsch R, Mayerhofer A (2005). The action of the mast cell product tryptase on cyclooxygenase-2 (COX2) and subsequent fibroblast proliferation involves activation of the extracellular signal-regulated kinase isoforms 1 and 2 (erk1/2). Cell Signal.

[50] Abe M, Kurosawa M, Ishikawa O, Miyachi Y, Kido H (1998). Mast cell tryptase stimulates both human dermal fibroblast proliferation and type I collagen production. Clin Exp Allergy.

[51] Tatler AL, Porte J, Knox A, Jenkins G, Pang L (2008). Tryptase activates TGFβ in human airway smooth muscle cells via direct proteolysis. Biochem Biophys Res Commun.

[52] Schubert TE, Weidler C, Borisch N, Schubert C, Hofstadter F, Straub RH (2006). Dupuytren’s contracture is associated with sprouting of substance P positive nerve fibres and infiltration by mast cells. Ann Rheum Dis.

[53] Borriello F, Granata F, Varricchi G, Genovese A, Triggiani M, Marone G (2014). Immunopharmacological modulation of mast cells. Curr Opin Pharmacol.

[54.••] Karkampouna S, Kruithof BP, Kloen P, Obdeijn MC, van der Laan AM, Tanke HJ (2014). Novel *ex vivo* culture method for the study of Dupuytren’s disease: effects of TGFβ type 1 receptor modulation by antisense oligonucleotides. Mol Ther Nucleic Acids.

[55] Karkampouna S, Kloen P, Obdeijn MC, Riester SM, van Wijnen AJ, Kruithof-de JM (2015). Human Dupuytren’s *ex vivo* culture for the study of myofibroblasts and extracellular matrix interactions. J Vis Exp.

[56.••] Mayerl C, Del Frari B, Parson W, Boeck G, Piza-Katzer H, Wick G (2016). Characterisation of the inflammatory response in Dupuytren’s disease. J Plast Surg Hand Surg.

[57] Crisan M, Yap S, Casteilla L, Chen C-W, Corselli M, Park TS (2008). A perivascular origin for mesenchymal stem cells in multiple human organs. Cell Stem Cell.

[58] Prockop DJ (2016). Inflammation, fibrosis, and modulation of the process by mesenchymal stem/stromal cells. Matrix Biol.

[59] Birbrair A, Zhang T, Wang Z-M, Messi Maria L, Mintz A, Delbono O (2015). Pericytes at the intersection between tissue regeneration and pathology. Clin Sci.

[60.••] Viil J, Maasalu K, Mäemets-Allas K, Tamming L, Lõhmussaar K, Tooming M (2015). Laminin-rich blood vessels display activated growth factor signaling and act as the proliferation centers in Dupuytren’s contracture. Arthritis Res Ther.

[61] Gao Y, Li M, Zhang X, Bai T, Chi G, Liu JY (2014). Isolation, culture and phenotypic characterization of human sweat gland epithelial cells. Int J Mol Med.

[62] Fiore EJ, Mazzolini G, Aquino JB (2015). Mesenchymal stem/ stromal cells in liver fibrosis: recent findings, old/new caveats and future perspectives. Stem Cell Rev.

[63] Álvarez D, Levine M, Rojas M (2015). Regenerative medicine in the treatment of idiopathic pulmonary fibrosis: current position. Stem Cells Cloning.

[64] Trial J, Entman ML, Cieslik KA (2016). Mesenchymal stem cell-derived inflammatory fibroblasts mediate interstitial fibrosis in the aging heart. J Mol Cell Cardiol.

[65] Wang Y, Chen X, Cao W, Shi Y (2014). Plasticity of mesenchymal stem cells in immunomodulation: pathological and therapeutic implications. Nat Immunol.

[66.••] Hindocha S, Iqbal SA, Farhatullah S, Paus R, Bayat A (2011). Characterization of stem cells in Dupuytren’s disease. Br J Surg.

[67.••] Iqbal SA, Manning C, Syed F, Kolluru V, Hayton M, Watson S (2012). Identification of mesenchymal stem cells in perinodular fat and skin in Dupuytren’s disease: a potential source of myofibroblasts with implications for pathogenesis and therapy. Stem Cells Dev.

[68.••] Ratkaj I, Bujak M, Jurišić D, Baus Lončar M, Bendelja K, Pavelić K, et al. Microarray analysis of Dupuytren’s disease cells: the profibrogenic role of the TGF-β inducible p38 MAPK pathway. Cell Physiol Biochem. 2012;30(4):927–42. **Microarray analysis indicated p38 MAPK pathway and CD90-stem cell markers as pathogenic factors in DD**. 10.1159/000341470 22965824

[69.••] Verhoekx JS, Mudera V, Walbeehm ET, Hovius SE. Adipose-derived stem cells inhibit the contractile myofibroblast in Dupuytren’s disease. Plast Reconstr Surg. 2013;132(5):1139–48. **This study suggested that adipose stem cells may deliver antifibrotic signals upon myofibroblasts instead of providing a source of (myo)fibroblasts**. 10.1097/PRS.0b013e3182a3bf2b 23924646

[70.••] Iqbal SA, Hayton MJ, Watson JS, Szczypa P, Bayat A. First identification of resident and circulating fibrocytes in Dupuytren’s disease shown to be inhibited by serum amyloid P and Xiapex. PLoS ONE. 2014;9(6):e99967. **Bone marrow derived monocytes differentiate into fibrocytes which are found for the first time in DD tissues; the differentiation of fibrocytes or their profibrotic properties are prone to inhibition when exposed to high levels of SAP and Xiapex collagenase treatment**. 10.1371/journal.pone.0099967 PMC4059720 24933153

